# Reduced Fertilization to Improve Sustainable Use of Resources and Preserve Postharvest Quality of Fresh-Cut Wild Rocket (*Diplotaxis tenuifolia* L.) in Soil-Bound and Soilless Cultivation

**DOI:** 10.3390/plants13040499

**Published:** 2024-02-10

**Authors:** Michela Palumbo, Lucia Bonelli, Bernardo Pace, Francesco Fabiano Montesano, Francesco Serio, Maria Cefola

**Affiliations:** 1 Institute of Sciences of Food Production, National Research Council of Italy (CNR), c/o CS-DAT, Via Michele Protano, 71121 Foggia, Italy; michela.palumbo@ispa.cnr.it (M.P.); bernardo.pace@ispa.cnr.it (B.P.); 2 Institute of Sciences of Food Production, National Research Council of Italy (CNR), Via G. Amendola, 122/O, 70126 Bari, Italy; lucia.bonelli@ispa.cnr.it; 3 Department of Soil, Plant and Food Sciences, University of Bari Aldo Moro, 70126 Bari, Italy; francesco.montesano@uniba.it

**Keywords:** *Diplotaxis tenuifolia* L., water use efficiency, nutrients productivity, ammonia content, respiration rate, electrolyte leakage

## Abstract

Reducing fertilizer input is a goal for helping greenhouse farming to achieve higher sustainability in the production process while preserving overall crop performance and quality. Wild rocket plants were cultivated in a plastic greenhouse divided into two independent sectors, one for soil-bound (SbS) cultivation and another equipped for soilless (ScS) cultivation systems. In both SbS and ScS, the crop was subjected to treatments consisting of a high- and a low-input fertilization program (HF and LF treatment, respectively). Water use efficiency (WUE) and partial factor productivity (PFP) for nutrients (N, P, K, Ca, and Mg for ScS, and N for SbS) were measured. Rocket leaves, separated for the cultivation system and fertilization program and collected at different cuts during the growing cycle, were cold stored at 10 °C until 16 d. On each sampling day (at harvest and during storage), the sensory parameters, respiration rate, dry matter, color, electrolyte leakage, antioxidant activity, total phenols, total chlorophyll and ammonia content were evaluated. In ScS, the PFP for all nutrients supplied as fertilizers showed a significant increase with the LF treatment, with values higher than 30% recorded for N, K, and Ca. As for the postharvest performance, rocket leaves cultivated in ScS showed better qualitative traits than those cultivated in SbS, as suggested by the lower values of ammonia content and electrolyte leakage recorded at the end of storage period in samples grown in ScS. Moreover, in ScS, the data showed lower membrane damage in LF than HF rocket leaves. Finally, regarding total chlorophyll content, even if no effect of each treatment was recorded in SbS, rocket cultivated in ScS showed a better retention of this parameter by applying LF rather than HF treatment. In addition to this, a PLS model (R^2^ = 0.7) able to predict the cultivation system, using as a variable non-destructively measured total chlorophyll content, was implemented. Low fertilization input, both in SbS and in ScS, allowed satisfying production levels and more sustainable management of nutrients. LF treatment applied to ScS also had in positive effects on the postharvest quality of fresh-cut rocket leaves.

## 1. Introduction

Increasing food production, sustained by the trend of growth in the world population, is causing considerable fertilizer consumption, aimed to balance the gap between the permanent removal of nutrients from the field, as a consequence of the harvested parts of the crop, and the natural availability of nutrients in the soil. This process raises questions about the sustainability of agricultural production, as the intensive use of natural resources and external inputs for agricultural production has a significant impact on the environment. The production of vegetable crops under controlled environments (i.e., greenhouses) has expanded considerably in recent decades in Mediterranean areas [1]. Initially, research efforts and the related introduction of technical innovations focused on high-quality and healthy products. However, concern with environmentally sustainable production has risen in the last few decades, as industrial greenhouse crops are usually seen as having a high environmental impact [2]. On the other hand, there is plenty of evidence that greenhouse vegetable production may decrease the environmental impact compared to field cultivation [3].

The efficient use of resources (water and fertilizers) is a target in irrigated greenhouse agriculture to achieve higher sustainability in the production process, better crop performance, and improved nutritional and sensorial quality [4,5]. Soilless cultivation produces several benefits compared to traditional systems, including the possibility to standardize the production process, improve plant growth and yield, and obtain higher efficiency in water and nutrient use [6]. Moreover, soilless cultivation allows us to overcome problems related to soil-borne pathogens and inadequate soil fertility [7].

Fertilization practice in vegetable production sector, with a particular focus on nitrogen (N), is under the spotlight [8]. In fact, over-fertilization has generally been perceived as a cheap insurance against yield loss, meaning that a general tendency to apply excessive nutrients, in particular N, is widespread, having an appreciable impact on the environment [9]. However, several factors, including consumers’ demand for sustainable production processes and regulatory pressure toward decreasing the negative impact of agriculture on the environment (e.g., Council Directive 91/676/EEC of 12 December 1991 concerning the protection of waters against pollution caused by nitrates from agricultural sources) [10], are promoting practices aimed at reducing the use of fertilizers.

According to trends observed in recent years, baby leaf vegetables such as spinach, rocket, and lettuce have gained economic importance since they are basic components of ready-made salads. Among these, wild rocket (*Diplotaxis tenuifolia* L.) is a popular leafy vegetable due to its distinct taste and nutritional content, and its cultivation is widespread and undergoing further expansion. Specifically, rocket leaves are rich in vitamins such as A, B, C, and K, iron, and essential proteins, which contribute to human health [11,12,13]. This product, much appreciated by consumers, is a good source of nutrients and antioxidant molecules, especially glucosinolates [14], but it is also a hyper-accumulator of nitrates [15].

As with most leafy vegetables, rocket leaves have high perishability during storage due to their high respiration rate [16], which limits their shelf-life. During storage, leaf quality rapidly decreases because of the increase in yellowing strictly related to chlorophyll degradation [17]. Usually, alterations in the common green color of leafy vegetables is a limiting factor for their marketability as they influence the consumer’s choice [18].

The quality of rocket leaves at harvest and during storage depends on several preharvest factors such as pedoclimatic factors, cultural practices (cultivation system, nutrient or water management), and genotype (landraces, cultivar) [15]. Among the cultivation strategies, soilless systems are considered efficient plant growing methodologies in the fresh-cut company, as it is possible to control the product quality by using the appropriate composition and management of nutrient solution [19]. Fertilization management can affect the quality parameters of leafy vegetables during storage, and at the market level, demand for products that are high quality in terms of having high organoleptic, nutritional, and functional properties is a crucial trait of the quality concept [20]. Although the reduction in fertilization levels is an object of considerable attention, specific research on wild rocket is limited, particularly if aimed at investigating the relationship between the fertilization level, cultivation system, and effects on product postharvest quality. In postharvest, it is necessary to start with a high-quality product to ensure a longer shelf-life.

The aim of this study is to evaluate the effects of different levels of fertilizers’ application in soilless (ScS) and soil-bound (SbS) cultivation systems in terms of inputs (water and nutrients), use efficiency and productivity, and the crop yield of rocket. Moreover, the effects of fertilization levels on quality traits at harvest and during postharvest were evaluated for rocket leaves produced by SbS or ScS, with the aim being to evaluate the feasibility of sustainable low-input fertilization for preserving quality at harvest and during postharvest.

## 2. Materials and Methods

### 2.1. Plant Material and Growing Conditions

Two parallel experiments, one in SbS and another in ScS cultivation conditions, were carried out at the Experimental Farm “La Noria” of the Institute of Sciences of Food Production (ISPA-CNR) in Mola di Bari, Italy (41°03′ N; 17°04′ E; 24 m above sea level), during the 2020–2021 growing season. Wild rocket (*Diplotaxis tenuifolia* L., cv. Dallas, Isi Sementi, Fidenza, Italy) plants were cultivated, under unheated plastic greenhouse conditions, into two independent environments, one equipped for SbS (a polyethylene multitunnel type) and another for ScS (a polymethacrylate rigid-panel type). In both cases, crop management practices (e.g., disease and pest control) were similarly based on local practices. In both cultivation systems, the crop was subjected to treatments consisting of high- and low-input fertilization programs named HF and LF, respectively (see Section 2.1.1 and Section 2.1.2 for specific details on the application of fertilization treatments in the two cultivation systems). Rockets seeds were sown on 14 October 2020 in polystyrene plug trays, and the seedlings were transplanted four weeks later with a density of 80 plant/m^2^ [7]. According to the common practice for wild rocket salad, the crop cycle consisted of subsequent harvest cuts and re–shooting cycles (in total, 6 were used in this experiment). In both cultivation systems, the treatments (LF and HF) were arranged in a complete randomized block design with five replications (320 plants per replication). The temperature ranged over the growing cycle between minimum and maximum values of 1.5 and 41.5 °C, respectively, with a mean temperature of 13.5 °C in the polymethacrylate rigid-panel type greenhouse, and between minimum and maximum values of 1.9 and 38.7 °C, respectively, with a mean temperature of 11.5 °C in the polyethylene multitunnel. Air relative humidity ranged between minimum and maximum values of 15.8 and 98.8%, respectively, with a mean humidity of 70.4% in the polymethacrylate greenhouse, and between minimum and maximum values of 21.9 and 94.1%, respectively, with a mean humidity of 64.6% in the polyethylene multitunnel.

#### 2.1.1. Soil-Bound Growing Conditions

In soil-bound sector (SbS), soil was a typical Mediterranean “Terra Rossa” clay soil. The main soil properties are reported in Table 1.

After transplant, plants were watered via drip irrigation, using rainwater stored in collection tanks. The irrigation schedule was based on local practices. Two fertilization treatments (SbS-HF and SbS-LF) were compared: 60 (HF) and 30 (LF) kg/ha of nitrogen, respectively, were applied for each cut and re-shooting cycle via fertigation as calcium nitrate. No other nutrients were applied because of the high P and K contents in the soil. The dose of each cut and re-shooting cycle was applied in two fertigation events. The fertigation doses were established based on a nitrogen dose recently tested in [7] for wild rocket (adopted in SbS-HF treatment) and a lowered dose based on previous experiments conducted by our team, suggesting that in the specific conditions of this trial, a 50% reduction in the dose would have been a sustainable choice.

#### 2.1.2. Soilless Growing Conditions

Seedlings were transplanted in 4.5 L plastic containers (20 plants per pot) filled with a peat/perlite (3:1 in volume) mixture (dry bulk density—0.12 g/cm^3^; total pore space—95%; air capacity—30%; maximum water holding capacity—65%; easily available water—25%, water buffer capacity—8%). Fertilizers were added to pre-collected rainwater for the preparation of the nutrient solution (NS). A drip irrigation system was adopted. The irrigation schedule, automatically operated using a timer, was subjected to periodical adjustments based on plant water need variations, assessed by measuring the leaching rate approximately every two days and using a <20% leaching fraction as a target. An open-cycle NS management approach was adopted. In the ScS–HF treatment, a standard NS [21] with slight modifications, commonly adopted in soilless cultivation, containing N (15.0 mM), phosphorus (1.0 mM), potassium (6.0 mM), magnesium (2.0 mM), calcium (5.0 mM), and sulfur (2.9 mM), was used. In the ScS–LF treatment, plants were fertigated with a NS with a reduced macro-nutrient concentration, as proposed in [7], containing N (11.0 mM), phosphorus (1.0 mM), potassium (4.4 mM), magnesium (1.7 mM), calcium (3.2 mM), and sulfur (2.1 mM). In both treatments, iron (20 μM), manganese (5 μM), zinc (2 μM), boron (25 μM), copper (0.5 μM), and molybdenum (0.1 μM) were added in NS as micronutrients.

### 2.2. Plant Growth, Dry Matter, Leaf Chlorophyll Content, Water Use Efficiency, and Nutrients Productivity

In the experiments, six cuts occurred from mid-December to early May. Harvests were carried out when leaves of different treatments reached the commercial harvest stage (approximately until several serrated 10–12 cm leaves were present on each plant) [22,23]. Leaf samples were collected and weighed in order to determine yields and placed into a forced-draft oven at 65 °C to determine the dry matter percentage.

On five leaves from each plot, leaf chlorophyll was measured using a rapid and non-destructive chlorophyll meter (Apogee MC-100, Apogee instruments, Logan, UT, USA) in January and February for SbS and February and March for ScS. Chlorophyll data obtained via this procedure were used to develop a partial least squares regression (PLSR) model for the prediction of a cultivation system using rapid and non-destructive tools (see Section 2.4 and Section 3.3).

Water use efficiency (WUE) was expressed as the fresh weight of edible product per liter of water supplied as irrigation. Partial factor productivity (PFP) for nutrients (N, P, K, Ca and Mg for ScS, and N for SbS) was expressed as the fresh weight of edible product per gram of nutrient supplied [24].

### 2.3. Postharvest Quality Parameters

The rocket leaves used during postharvest storage were harvest cut during the months of January and February and March for SbS and ScS, respectively. At two consecutive harvest cut times (HC1 on 10 February and HC2 on 3 March), fresh-cut rocket leaves, separated per the cultivation conditions and fertilization program, were transported in refrigerated condition to the postharvest laboratory of ISPA-CNR in Foggia for the analysis of postharvest quality. The rocket leaves with defects and mechanical damage were removed, while good samples were packed in open polyethylene bags (Orved, Musile di Piave, Italy), with about 600 g of sample in each one. In particular, 10 bags (5 replicates × 2 fertilization programs) were prepared for each cultivation condition, with a total of 20 PP bags. Subsequently, all samples were stored at 10 (±1) °C (as commonly occurs in the Italian retailer market) for 16 d. On each sampling day (at harvest and after 6, 9, 12, and 16 days of storage), sensory parameters (visual quality and odor), respiration rate, dry matter, color parameters, electrolyte leakage, antioxidant activity, total phenols, total chlorophyll, and ammonia content were evaluated.

#### 2.3.1. Respiration Rate, Dry Matter, Electrolyte Leakage, and Ammonia Content

The respiration rate of fresh-cut rocket leaves was determined at 10 °C at harvest and at each sampling time using a closed system, as reported in [25], albeit with slight modifications. In detail, amounts of about 100 g for each replicate of rocket leaves were taken from each bag for analysis and put into a 3.6 L sealed plastic jar, where CO_2_ was allowed to accumulate up to 0.1% as the concentration of the CO_2_ standard. The time taken to reach this value was detected by taking CO_2_ measurements at regular time intervals [26]. The respiration rate was expressed as µmol CO_2_/kg s.

After the respiration rate analysis, the same samples were used for postharvest destructive determinations, as described below.

The dry matter content was calculated as the percentage ratio between the dry and fresh weights of rocket leaves. To determine the dry weight, 30 g per replicate of chopped fresh-cut rocket leaves was dried using a forced ventilation oven (M700-TB, MPM Instruments, Bernareggio, Italy) at 65 °C until reaching a constant mass.

Electrolyte leakage was evaluated by following the methodology reported in [25]. Rocket leaves’ disks, obtained using a cork borer for about 2.5 g per replicate, were dipped in 25 mL of distilled water. After 30 min of storage at 10 °C, the conductivity of the solution was measured via a conductivity meter (Cond. 51+—XS Instruments, Carpi, Italy). After that, the tubes with leaf disks were frozen for 48 h. Successively, on the samples thawed, the conductivity was measured, and the same was calculated as the percentage ratio of initial over total conductivity.

Ammonia content was evaluated according to the method of [27]. About 5 g of chopped rocket leaves were homogenized for 2 min in 20 mL of distilled water in cold conditions and then centrifuged for 5 min at 6440× *g* at 4 °C. Then, the supernatant (0.5 mL) was mixed with 5 mL of nitroprusside reagent (phenol and hypochlorite in alkali reaction mixture) and heated at 37 °C for 20 min. The color development after incubation was determined using the spectrophotometer (UV-1800, Shimadzu, Kyoto, Japan) (reading the absorbance at 635 nm). The content of NH_4_^+^ was expressed as μg NH_4_^+^/g of fresh weight (fw), using ammonium sulphate as the standard (0–10 μg/mL, R^2^ = 0.999).

#### 2.3.2. Sensory Analysis and Color Parameters

During storage, regarding sensory visual quality (VQ), samples were taken from each PP bag and evaluated by a group of 6 trained researchers (made up of 2 female and 4 male panelists) using a 5 to 1 rating scale proposed in [25], where 5 = very good, 4 = good, 3 = fair, 2 = poor, and 1 = very poor. A score of 3 was considered to be the shelf-life limit, while a score of 2 represented the limit of edibility.

The CIELab color parameters (L*, a* and b*) were measured, for each replicate, on 3 random points on the surface of 10 fresh-cut rocket leaves using a colorimeter (CR400, Konica Minolta, Osaka, Japan). The calibration of the instrument was performed with a standard reference, with 97.44, 0.10, and 2.04 as the values of L*, a*, and b*, respectively. To measure color variations during storage, ΔE* was calculated according to the equation reported in [28], while the hue angle was obtained via the equations reported in [29]. Additionally, the yellowness index (YI) was calculated from primary L*, a*, and b* readings, according to the equation reported in [29]:

(1) $$\mathrm{YI}=\frac{\left(142.86\times b*\right)}{L*}$$

#### 2.3.3. Antioxidant Activity, Total Phenols, and Total Chlorophyll Content

The same extraction procedure was used for the DPPH assay and Folin–Ciocalteu reducing capacity analysis, as reported in [30]. In detail, for each replicate, 5 g of chopped rocket leaves was homogenized in 20 mL methanol/water solution (80:20 *v*/*v*) for 2 min, using a homogenizer (T-25 digital ULTRA-TURRAX^®^—IKA, Staufen, Germany), and then centrifuged (Prism C2500-R, Labnet, Edison, NJ, USA) at 6440× *g* for 5 min at 4 °C. The extracts were collected and stored at −20 °C until the analysis.

The antioxidant activity was measured according to the procedure of the DPPH assay described in [31] using the methanol extracts. The absorbance at 515 nm was read after 40 min using a spectrophotometer (UV-1800, Shimadzu, Kyoto, Japan). The results were expressed as milligrams of Trolox per 100 g of fw using a Trolox calibration curve (82–625 µM; R^2^ = 0.999).

The total phenol content was evaluated according to [32]. In detail, 100 µL of each extract was mixed with 1.58 mL of water, 100 µL of the Folin–Ciocalteu reagent, and 300 µL of sodium carbonate solution (200 g/L). The absorbance at 765 nm was detected after 2 h of incubation in the dark, and the results were reported as milligrams of gallic acid equivalent (GAE) per 100 g of fw. The calibration curve of gallic acid was prepared with five points, from 50 to 500 µg/mL, and R^2^ = 0.998.

The total chlorophyll content was measured using the spectrophotometric method reported in [33]. In detail, 5 g of chopped samples was extracted in 50 mL acetone/water (80:20 *v*/*v*) solution using the homogenizer for 1 min and then centrifuged (SL 16 Centrifuge—Thermo Fisher Scientific, Langenselbold, Germany) at 6440× *g* for 5 min. The extraction was repeated 5 times (10 mL of acetone/water solution per time) to remove all pigments, and the extracts were combined. The absorbance was read on extracts properly diluted at three wavelengths (663.2 nm, 646.8 nm, and 470 nm). The total chlorophyll content was expressed as milligrams per 100 g of fw using the equation reported in [34].

### 2.4. Statistical Analysis

Plant growth parameters, WUE, and nutrients’ PFP data were subjected to analysis of variance (ANOVA). Treatment means were considered different when there was a significant effect at the *p* < 0.05 level. The statistical software STATISTICA 10.0 (StatSoft, Tulsa, OK, USA) was used for the analysis.

For postharvest quality parameters, a two multifactor ANOVA for *p* ≤ 0.05 was performed with the aim of evaluating the effects of the fertilization programs (HF or LF), the storage times, and their interactions on the postharvest quality parameters of fresh-cut rocket leaves cultivated in SbS or ScS. The mean values (n = 5) were separated using the Student–Newman–Keuls (SNK) test (*p* ≤ 0.05). The statistical software Statgraphics Centurion (version 18.1.12, Warrenton, VA, USA) was used for the analyses.

Partial least squares regression (PLSR) was run using the software The Unscrambler X (CAMO AS, Oslo, Norway) in order to develop and compare models able to predict the cultivation system, using as predictors chlorophyll data obtained via the Apogee meter or through the conventional destructive methodology.

## 3. Results and Discussion

### 3.1. Water Consumption, Crop Performance, Plant Physiology, and Chemical Composition

The ScS improved earliness compared to SbS (the HC1 in ScS occurred on 21 December, while in SbS, a 17-day delay was observed). However, with the progress of the production cycle, the time gap between harvests in the two systems gradually narrowed. This may be due to the optimal growing conditions provided to plants by soilless cultivation, leading to reduced transplant stress, as also outlined for other species [35].

However, it should be noted that soil cultivation could also imply the presence of advantageous growing conditions in some cases. Di Gioia et al. [7] observed higher yield of rocket in soil compared to soilless cultivation due to better water availability conditions. Moreover, the root environment temperature can be sub-optimal in soilless cultivation in unheated greenhouse conditions [36].

In ScS, the cumulative yield and the dry matter percentage of the ScS-HF were higher than for the ScS-LF treatment (by 11 and 10%, respectively—Table 2). On the other hand, WUE was slightly lower in ScS-HF compared to ScS-LF (Table 2), and a more remarkable effect was observed on PFP, which was 30, 31, 8, 40, and 21% lower for N, K, P, Ca, and Mg, respectively, in ScS-HF compared to ScS-LF (Table 2). The PFP parameter represents a simple but effective expression of production efficiency in terms of the units of harvested product per unit of nutrients applied as fertilizer, and it was clearly higher with low-fertilization conditions.

Additionally, in SbS, the cumulative production and the dry matter percentage observed in SbS-HF treatment were higher than for the SbS-LF treatment (by 9 and 4%, respectively—Table 3). Contrary to what was observed in ScS, where only slight effects on WUE were observed, in SbS-HF, the WUE was 9.2% higher than in SbS-LF, while the N PFP was higher in the SbS-LF (by 84%).

Nutrient availability is an essential factor for maximizing production, and it is well known that rocket responds well to good nutrient availability in the root zone [35,37,38]. The different effects of treatments on WUE in SbS (more pronounced) and ScS (slighter) growing conditions can be explained by the possibility offered by soilless cultivation for the relatively easy optimization of irrigation management compared to soil-bound growing conditions in the absence of advanced methods for irrigation management (sensors, ET estimation models). In fact, measuring leaching and adjusting irrigation scheduling based on a target leaching rate value may theoretically contribute to preventing, at a certain extent, excessive irrigation. However, it should be recognized that, under a practical point of view, the use of timer with prefixed irrigation schedule subjected to periodical adjustments (generally weekly or on a higher time-span basis) based on leaching measurements is generally inefficient because it does not take into account the changes in real plant water consumption occurring on a certainly shorter time-scale (not very compatible with the practical cultivation operations of a standard Mediterranean farm).

The PFP has always been far higher with LF treatments, both in soil-bound and soilless conditions, allowing for both excellent production levels and more sustainable management of nutrients. The effects of fertilization on product quality should also be taken into account, in particular in rocket salad, with this species being a hyper-accumulator of nitrates [39]. In a previously published paper related to the experiment presented in this manuscript [23], it was reported that the fertilization dose and the growing system did not modify nitrate content, with the exception of the soilless system treated with the highest fertilizer dose, where nitrate slightly exceeded the regulatory limits. In this context, the correct management of the fertilizer application represents one of the major challenges to be faced for the rational use of inputs in agriculture, as well as for maintaining good production levels while minimizing environmental impact, as required by the increasingly pressing demands of consumers, who want more sustainable production processes [8,40]. In both ScS and SbS, the leaf chlorophyll content was not influenced by the levels of fertilizer application (Table 2 and Table 3).

### 3.2. Postharvest Quality Parameters

#### 3.2.1. Respiration Rate, Sensory VQ, and Color Parameters

In Table 4, the effects of the fertilization programs (HF or LF), storage times (0, 6, 9, 12 or 16 days), and their interactions on the sensory, physical, and chemical parameters of fresh-cut rocket leaves stored at 10 °C and cultivated on ScS or SbS are reported. As expected, results from the multifactor ANOVA showed that the storage time affected all parameters analyzed in SbS and ScS, except for the dry matter (Table 4). The main effects of the storage time on quality postharvest parameters are well known. It is widely demonstrated that during postharvest storage, vegetables produce heat, water vapor, and CO_2_, which cause physiological and chemical changes, with the consequent postharvest degradation of the product [41].

**Figure 1:**
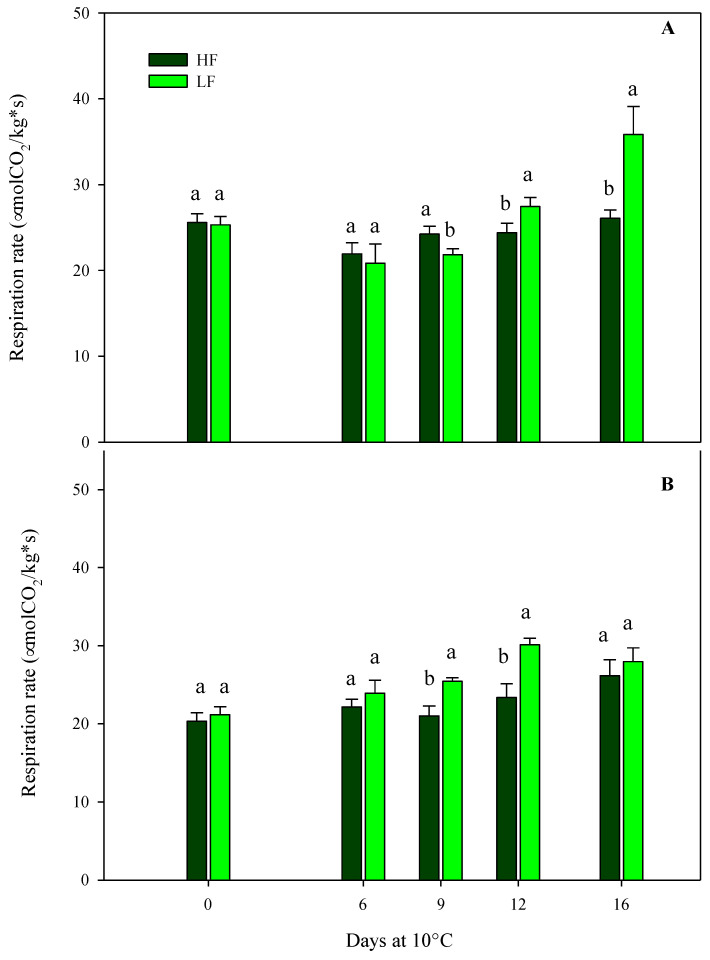
Changes in the respiration rates of fresh-cut rocket leaves sampled at two subsequent harvest-cut times during the growing cycle [harvest cut 1 (**A**) and 2 (**B**)], cultivated via a soilless system (ScS) treated with different fertilization programs (high input, HF, or low input, LF) and stored for 16 days at 10 °C. Data are the means of five replicates ± standard deviation. Within the same storage time, different letters (a, b) indicate statistical differences (*p* ≤ 0.05), according to the SNK test.

Regarding the respiration rates of samples cultivated on ScS, the results obtained from the multifactor ANOVA showed that all factors (fertilization programs, storage times, and their interactions) were significant in the two harvest cuts (Table 4). At harvest, no statistical differences were observed among treatments at both harvest times, showing high respiration values [26] in the range of 20–36 µmol CO_2_/kg s, in agreement with the values reported in [25] (Figure 1). Other authors reported values of respiration rates in rocket leaves stored at 4 °C or 17 °C of about 8.5 and 32.8 μmol CO_2_/kg s, respectively [42,43]. These differences could be due to the effects of cultivars, preharvest management practices, climatic conditions, the maturity stages of the leaves at harvest, storage temperatures, and postharvest handlings. Optimal postharvest conditions, including storage times and temperature management, can slow down the biological processes caused by senescence and maturation, reducing or inhibiting the development of physiological disorders, such as the increment in the respiration rate [11,44].

In HC1, the trend of the respiration rate in ScS-HF samples was almost the same after 16 days, while ScS-LF samples showed an increase (41.6% respect to fresh samples) from the 12th day until the end of storage (Figure 1A). Regarding the HC2, the ScS-LF rocket leaves started to show higher values of respiration rate compared to ScS-HF samples from the 9th day of storage, reaching the highest rate on the 12th day (30.1 ± 0.86 µmol CO_2_/kg s). Afterward, it remained almost constant until the end of storage, showing no statistical differences among the fertilization programs (Figure 1B). Differences in respiration rate among the fertilization treatments could be related to the dry matter content in HC1. Ref [45] reported that wild rocket with high respiration rates had higher dry matter contents than leaves with lower respiration rates.

Regarding the results of the multifactor ANOVA performed on rocket leaves cultivated on SbS, the respiration rate was only affected by the storage time in both harvest cuts, showing mean values of about 36.7 µmol CO_2_/kg s at harvest (Table 4).

**Figure 2:**
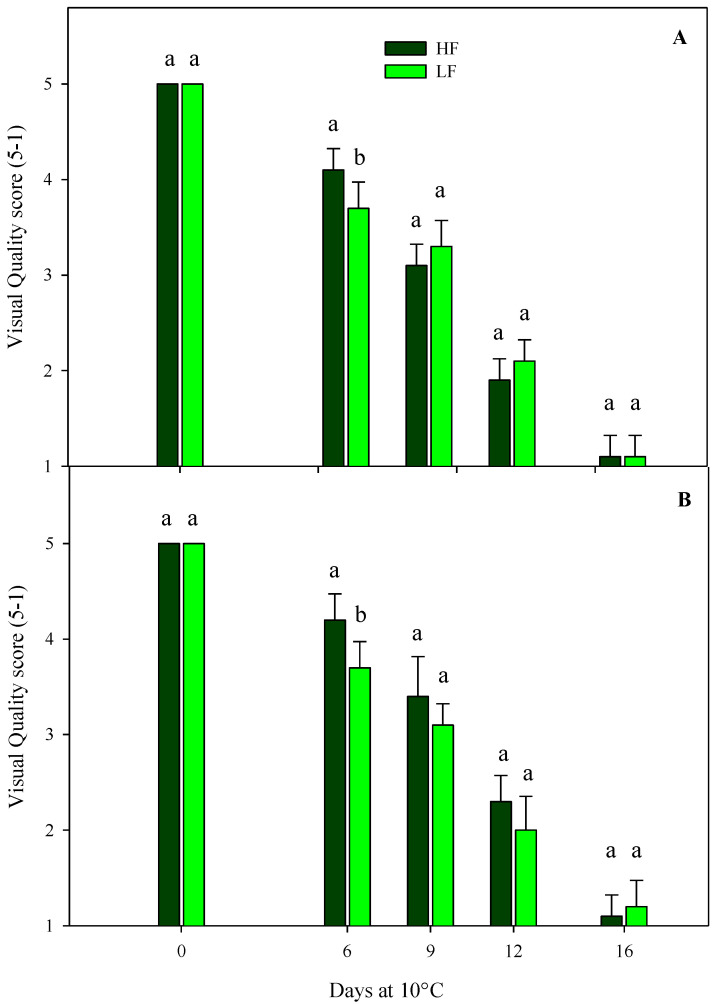
Changes in the sensory visual quality (VQ) scores of fresh-cut rocket leaves sampled at two subsequent harvest-cut times during the growing cycles [harvest cut 1 (**A**) and 2 (**B**)], cultivated via a soilless system (ScS) treated with different fertilization programs (high-input, HF, or low-input, LF) and stored for 16 days at 10 °C. A 5 to 1 rating scale was considered, where 5 = very good, 4 = good, 3 = fair, 2 = poor, and 1 = very poor. A score of 3 represents the shelf-life limit, while a score of 2 is considered the limit of edibility. Data are the means of five replicates ± the standard deviation. Within the same storage time, different letters (a, b) indicate statistical differences (*p* ≤ 0.05), according to the SNK test.

Regarding the sensory VQ of rocket leaves cultivated on ScS, in both harvest cuts, all treatments showed a gradual decrease during the storage, reaching the shelf-life limit (score 3) and the limit of edibility (score 2) after 9 and 12 days, respectively (Figure 2). Slight significant differences were observed among the fertilization treatments, probably due to the variability in the samples. These results demonstrate that it is possible to reduce the quantity of fertilizer during cultivation without compromising the visual aspect of the fresh-cut rocket leaves, with positive impacts on consumers’ choices that are more sensitive to the sustainability and environmental impacts of production approaches [25]. The sensory VQ of rocket leaves obtained by SbS was significantly affected by the storage time in both harvest cuts (Table 4). As expected, a decrease was observed during the storage, reaching the commercial limit (score 3) and the limit of edibility (score 2) after 12 and 16 days, respectively.

**Figure 3:**
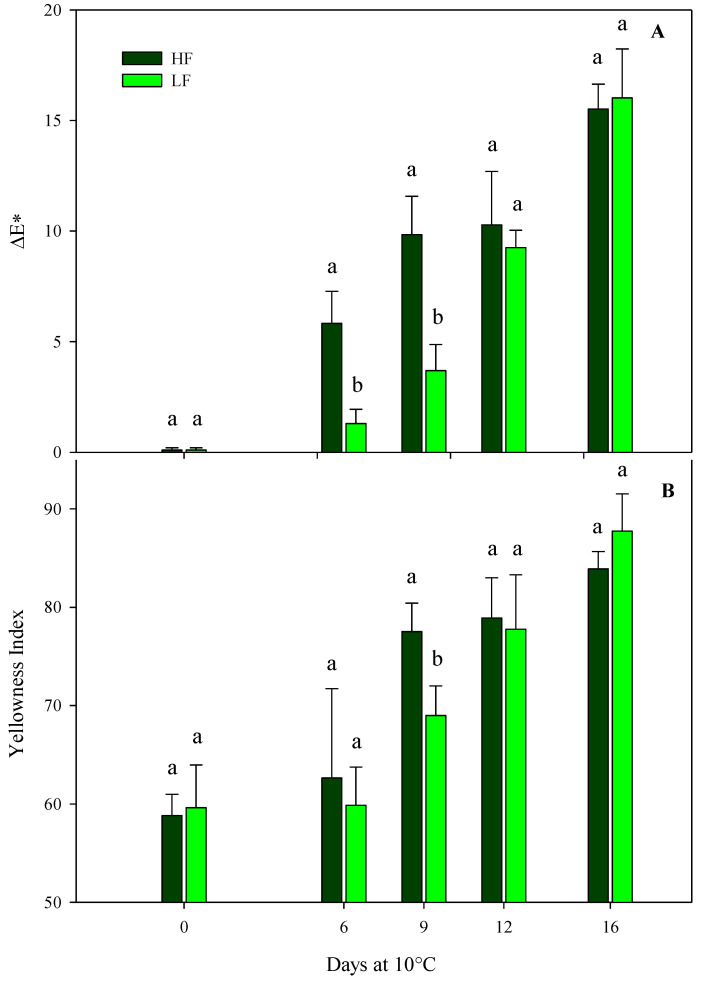
ΔE* (**A**) and yellowness index (**B**) of fresh-cut rocket leaves sampled at harvest cut 1 cultivated via a soilless system (ScS) treated with different fertilization programs (high input, HF, or low input, LF) and stored for 16 days at 10 °C. Data are the means of five replicates ± standard deviation. Within the same storage time, different letters (a, b) indicate statistical differences (*p* ≤ 0.05), according to the SNK test.

In green leafy vegetables, a loss of VQ and a reduction in the consumer’s acceptability are strictly associated with a reduction in green color during postharvest storage. Indeed, color is one of the most important traits for food preference and pleasantness, and it determines the freshness of rocket leaves [46]. ΔE* and YI were considered in this research work to be significantly influenced by the interaction of the factors at HC1, while the hue angle was affected only by the storage time (Table 4), demonstrating a gradual yellowing of rocket leaves during storage. Indeed, after just 6 days, ΔE* was significantly higher in the ScS-HF sample than the ScS-LF leaves, showing values about 4-fold higher (Figure 3A). The trend after 9 days of storage was almost the same, while an increment in ΔE* was recorded in ScS-LF rocket leaves on the 12th and 16th days, with no statistical differences between treatments. Regarding the hue angle, a slight decrease was recorded during the entire storage period, with no statistical differences between the fertilization treatments, showing rocket leaves to be more yellowish compared to the ones at harvest (Table 4) [47].

The results for ΔE* were confirmed by the YI that verified the loss of green color in rocket leaves in line with the storage time (Figure 3B). In particular, YI recorded a gradual increase for both the fertilization treatments during the storage time and showed the highest significant difference among treatments at day 9 (values of 77.5 ± 2.9 and 68.9 ± 3.0 in ScS-HF and ScS-LF samples, respectively). Until the end of storage period, YI values continued to increase, with no statistical differences between treatments (Figure 3B). Regarding the HC2, ΔE* and the hue angle were only influenced by the storage time, while YI was also affected by the fertilization program (Table 4). ScS-HF samples showed mean values of YI higher (about 2.5%) than those of ScS-LF rocket leaves (70.2 ± 1.2). All the results for the color parameters of rocket leaves cultivated in ScS during both harvest cuts support the hypothesis that higher fertilization could cause a more rapid loss of color than lower strategies, probably due to the greater perishability of the high-fertilized product. As above reported, during postharvest storage, the retention of green color in green leafy vegetables is strictly related to the VQ and consumers’ preferences, and our results underline the importance of adequate nutrition for obtaining rocket leaves with optimal color [19,48].

As for rocket leaves cultivated on SbS, results from the multifactor ANOVA showed that all color parameters of rocket leaves were only affected by the storage time for both harvest cuts (Table 4). In detail, the storage time caused an increase in the ΔE and YI values and a decrease in the hue angle in the HC1 and the HC2, confirming a gradual yellowing of rocket leaves.

#### 3.2.2. Ammonia Content, Electrolyte Leakage, and Dry Matter

**Figure 4:**
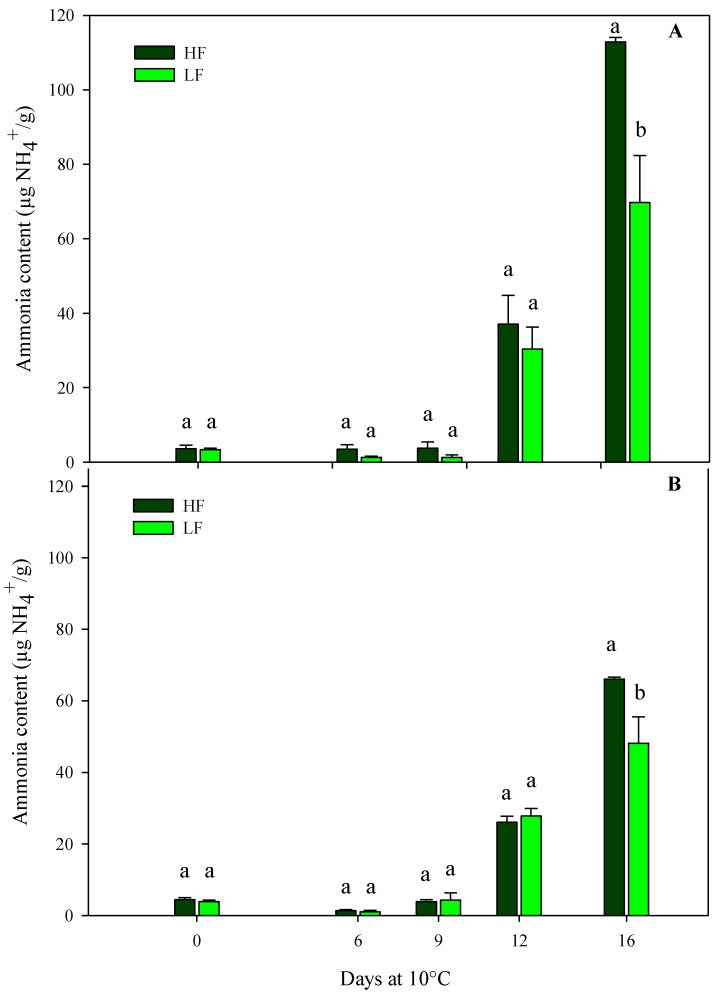
Changes in ammonia content in the harvest cut 1 (**A**) and 2 (**B**) of fresh-cut rocket leaves cultivated via a soilless system (ScS) treated with different fertilization programs (high input, HF, or low input, LF) and stored for 16 days at 10 °C. Data are the means of five replicates ± standard deviation. Within the same storage time, different letters (a, b) indicate statistical differences (*p* ≤ 0.05), according to the SNK test.

The results from the sensory VQ were confirmed using the physiological parameters (ammonia content, electrolyte leakage, and dry matter). Regarding the ammonia content, in both harvest cuts of rocket leaves obtained by ScS, it was influenced by all factors (fertilization programs, storage times, and their interactions) (Table 4). In detail, ammonia started to accumulate on the 12th day of storage and reached higher values in ScS-HF samples than in ScS-LF at the end of the storage time in both harvest cuts (Figure 4). In contrast, in rocket cultivated on SbS, the ammonia content was only affected by the storage time in both harvest cuts (Table 4). These differences between fertilization treatments might be explained by considering the data on chlorophyll degradation reported below. In detail, ammonia accumulation is the result of chlorophyll catabolism, which induces the degradation of apoproteins by protease and the remobilization of the nitrogen of the chlorophyll apoproteins [49]. Moreover, the results on ammonia content are in agreement with the hue angle values observed during storage and suggest the existence of a correlation between these two parameters in leafy vegetables [50]. All these results confirm that the ammonia content might be considered an objective indicator of the senescence and marketability of leafy vegetables [51]. In detail, its accumulation in vegetable tissues is associated with the senescence of the leaves, and high concentrations of this component are responsible of tissue damages, which lead to the senescence and have a negative impact on the overall quality of the product. Moreover, in minimally processed products, like green leafy vegetables, injuries that occur during postharvest handlings activate degradation processes like proteolysis, causing the release and accumulation of ammonia [13,52].

**Figure 5:**
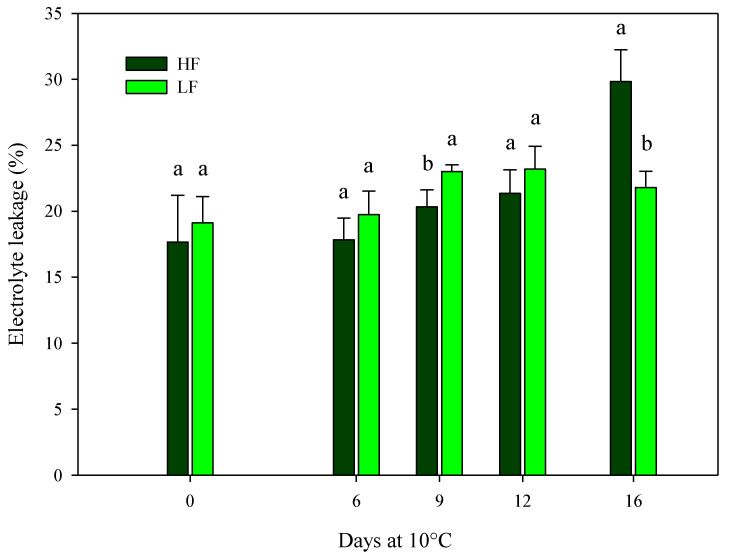
Changes in the electrolyte leakage of fresh-cut rocket leaves sampled at harvest cut 2 cultivated via a soilless system (SS) treated with different fertilization programs (high input, HF, or low input, LF) and stored for 16 days at 10 °C. Data are the means of five replicates ± standard deviation. Within the same storage time, different letters (a, b) indicate statistical differences (*p* ≤ 0.05), according to the SNK test.

The findings for ammonia content are in agreement with the ones recorded for electrolyte leakage, indicating the cell membrane integrity. On samples cultivated in ScS, the electrolyte leakage was only affected by the fertilization program and the storage time in HC1 and by all factors (fertilization programs, storage times, and their interactions) in HC2 (Table 4). Considering the HC1, higher significant values were observed in ScS-HF fresh-cut rocket leaves (22.9 ± 2.4%) compared to the ScS-LF samples (18.4 ± 1.1%). In the HC2, the values of electrolyte leakage were almost constant along the entire storage time for ScS-LF samples, while the ScS-HF leaves showed the highest value at the end of storage (29.8 ± 2.4%) (Figure 5). Electrolyte leakage is strictly related to the quality and shelf-life of fresh-cut leafy vegetables, and it is commonly used to measure the integrity of cell membranes damaged by oxidative stresses in fresh-cut tissues [13,53]. Since the electrolyte leakage can be considered another indirect measure of plant senescence, expressed as the integrity of the cell membrane [28] together with the ammonia content, the results indicate a positive effect of the ScS-LF strategy on the reduction in membrane damage during postharvest storage. Similar results were reported in [13] for fresh-cut rocket leaves, where the percentage increase in electrolyte leakage along the storage on soil system was higher than the one detected on soilless system, pointing out that the soilless system may be more efficient in terms of the reduction in induced oxidative stresses on cell membranes.

Based on the ANOVA results on rocket cultivated on SbS, the electrolyte leakage was only affected by the storage time in both harvest cuts (Table 4), showing a gradual increase in line with the postharvest phase. The findings on ammonia content and electrolyte leakage on rocket leaves cultivated in SbS and ScS suggest the higher efficiency of the soilless approach than the soil one for controlling mineral nutrition of vegetables [54]. In this research paper, rocket leaves cultivated in ScS showed better qualitative performances for the postharvest storage than those in SbS, as indicated by the lower values of ammonia content and electrolyte leakage recorded at the end of storage in samples grown in ScS.

The results regarding electrolyte leakage are confirmed by dry matter values, as reported in Table 4. In the HC1 of ScS, dry matter content was affected by the fertilization program. In detail, ScS-LF samples had higher values (+21.8%) of dry matter than ScS-HF leaves (11.60 ± 1.24%). It is well documented that higher levels of fertilization, especially excesses of nitrogen, significantly contribute to vegetable overgrowth and biomass development, as well as to the reduction in the dry matter content [55,56]. This parameter plays an important role in the sensory VQ and consumer acceptability of leafy vegetables as it gives mechanical strength to vegetable tissues. Additionally, these results regarding dry matter confirmed the results obtained for the respiration rate: rocket leaves with high dry matter content showed higher respiration rates than the ones with lower dry matter [45].

#### 3.2.3. Chlorophyll Content, Antioxidant Activity, and Total Phenols

**Figure 6:**
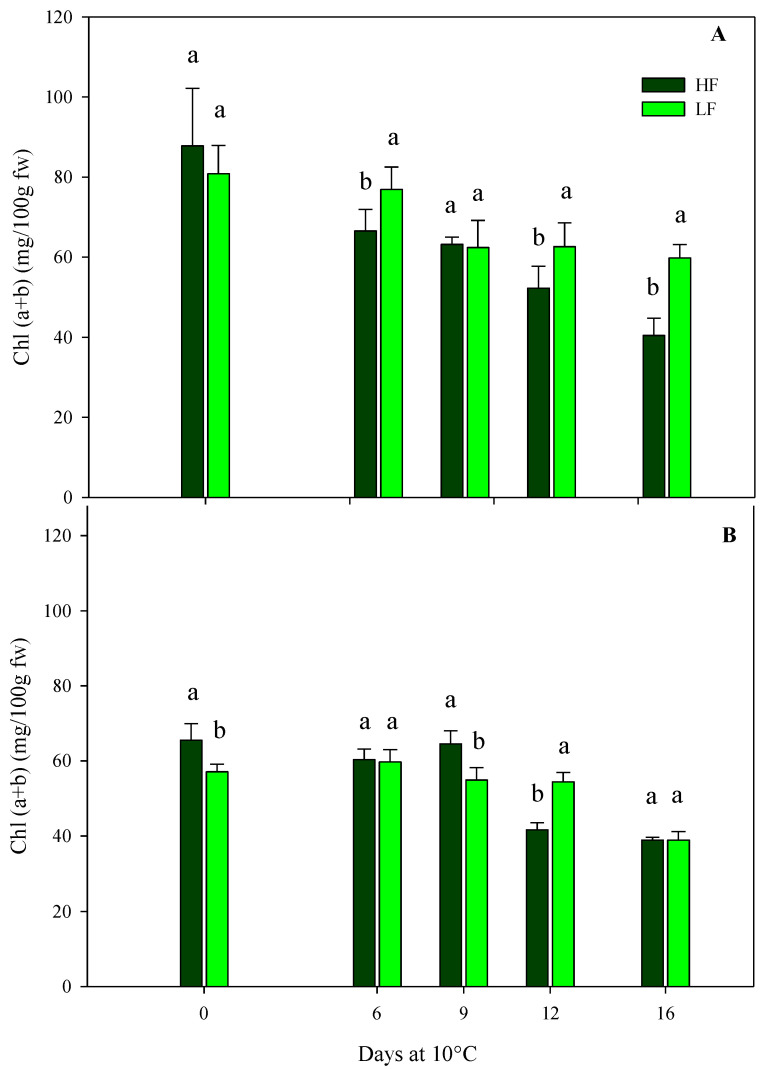
Effects of fertilization programs (high input, HF, or low input, LF) on the total chlorophyll contents of fresh-cut rocket leaves sampled at two subsequent harvest-cut times during the growing cycle [harvest cut 1 (**A**) and 2 (**B**)], cultivated on soilless system (ScS) and stored for 16 days at 10 °C. Data are the means of five replicates ± standard deviation. Within the same storage time, different letters (a, b) indicate statistical differences (*p* ≤ 0.05), according to the SNK test.

The total chlorophyll content of rocket leaves confirmed the results of the sensory and color parameters. In general, in leafy vegetables, the loss of the green color during postharvest storage through chlorophyll breakdown is one of the most important qualitative traits that influences the shelf-life [13]. As for samples obtained by ScS, in the case of HC1, the initial chlorophyll content was about 84.29 (±10.73) mg/100 g fw, with no statistical differences between treatments (Figure 6A). During storage, the chlorophyll values experienced significant reductions due to HF and LF treatments. In particular, at the end of storage, ScS-HF leaves showed a reduction in chlorophyll content, reaching a 2-fold fall compared to fresh samples; instead, in ScS-LF rocket leaves, these values decreased until the 9th day and, afterward, remained almost constant until the end of storage (Figure 6A). In the subsequent harvest cut (HC2), the initial chlorophyll content in both ScS-HF and ScS-LF samples was lower than the one measured in HC1 (−32%), and it remained almost the same until the 9th day, without significant differences between the fertilization treatments (Figure 6B). After 12 days, the lowest value was recorded in ScS-HF samples (41.65 ± 1.91 mg/100 g fw), while only after 16 days was a reduction in this parameter recorded for ScS-LF rocket leaves. Clearly, the differences in chlorophyll content were consistent with the differences in leaf color described before, being lower and decreasing faster in the ScS-HF samples than the ScS-LF samples during the storage of HC1 and HC2, respectively. Moreover, these results are in agreement with the ones obtained for ammonia content. Many research works have proved the influence of preharvest factors on the postharvest quality of leafy vegetables, and fertilization is one of them [57]. Additionally, at harvest, rocket leaves had a bright green color, but during postharvest, they became yellow, with a general loss of visual quality [58], involving many enzymatic reactions such as chlorophyllase activity. Moreover, chlorophyll breakdown also took place when a physiological stress occurs on the tissues, such as the mechanical stress induced via cutting at harvest [46], as was also demonstrated by the increase in electrolyte leakage in ScS-HF rocket leaves, as reported above.

In SbS, the chlorophyll content of rocket leaves was not influenced by the fertilization treatments but only by the storage time in both harvest cuts (Table 4), showing a significant reduction in line with the postharvest phase as a consequence of the senescence of leaves.

The ScS-LF strategy has a positive effect on the reduction in leaf yellowing at harvest and during postharvest storage.

**Figure 7:**
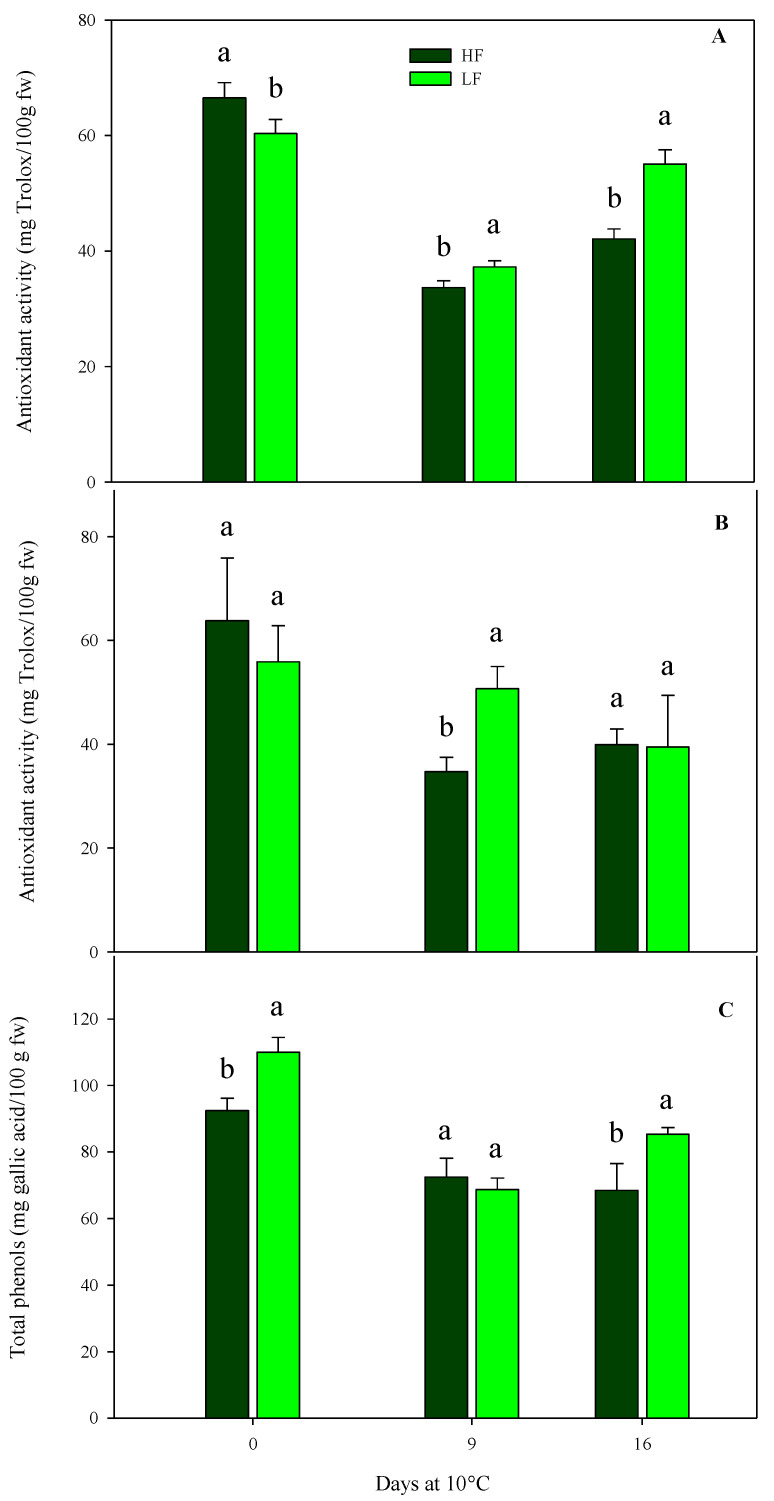
Effects of fertilization programs (high input, HF, or low input, LF) on the antioxidant activity of fresh-cut rocket leaves sampled at two subsequent harvest-cut times during the growing cycle [harvest cut 1 (**A**) and 2 (**B**)], cultivated via a soilless system (ScS) and stored for 16 days at 10 °C. Subfigure (**C**) reports changes in total phenols at HC1. Data are the means of five replicates ± standard deviation. Within the same storage time, different letters (a, b) indicate statistical differences (*p* ≤ 0.05), according to the SNK test.

As for chemical parameters in rocket leaves cultivated on ScS, antioxidant activity was significantly affected by all factors in both harvest cuts, while the total phenol content was influenced by all factors in HC1 and only by the storage time in the HC2 cut (Table 4). In detail, the initial antioxidant activity leaf content was about 61.6 (±6.0) mg Trolox/100 g fw in both harvest cuts, with no statistical differences between fertilization treatments (Figure 7). Subsequently, in the HC1, the antioxidant activity recorded the highest value in ScS-LF rocket leaves (55.1 ± 2.5 mg Trolox/100 g fw) at the end of storage (Figure 7A). As for the HC2, upon comparing treatments on the 9th day, significant differences were observed between the ScS-HF samples and ScS-LF ones, which showed the lowest (34.70 ± 2.83 mg Trolox/100 g fw) and the highest (50.68 ± 4.29 mg Trolox/100 g fw) values of antioxidant activity, respectively (Figure 7B).

A similar trend was observed for total phenol content in the case of HC1 of ScS (Figure 7C). The content at harvest was higher in ScS-LF rocket leaves than in ScS-HF samples and decreased in both treatments until the 9th day, with no significant differences. At the end of storage, the highest value of total phenols was observed in ScS-LF leaves (85.30 ± 2.02 mg gallic acid/100 g fw). These results prove the existence of a positive correlation between total phenols and antioxidant activity, as already reported for many fruit and vegetables [30,59], confirming that phenols highly contribute to the antioxidant activity of the product. In the present study, a significant increase in phenolic compounds was induced by low concentrations of nutrients in accordance with [23], which reported similar results for rocket leaves cultivated in a soilless system. This increment could be explained by the theory of the protein competition model reported in [60], according to which higher biomass accumulation and secondary metabolites are inversely correlated. In detail, protein synthesis and phenolic biosynthetic pathways use the phenylalanine as the same precursor. Thus, the phenylalanine is preferentially conveyed toward protein synthesis rather than the phenylpropanoid pathway when there is an increased protein request.

In rocket leaves obtained with SbS, antioxidant activity was significantly influenced by the two factors (fertilization program and storage time) in the HC1, and only by the storage time in the HC2 (Table 4). Comparing the fertilization treatments, in the HC1 differences were observed between SbS-HF (77.5 mg Trolox/100 g fw) and SbS-LF samples (83.5 mg Trolox/100 g fw). Finally, results obtained from the multifactor ANOVA showed that the total phenol content was only affected by the storage time in the HC1, while no significant influences were observed in the case of HC2 (Table 4).

### 3.3. Prediction of Cultivation System of Rocket Leaves Using Rapid and Non-Destructive Tools

Two PLS models were built in order to predict the cultivation system. In the first PLS (PLS-1), the hue angle and the total chlorophyll measured using the non-destructive tool were considered to be predictors. In the second PLS (PLS-2), the x variables were the hue angle and the total chlorophyll measured using the analytic method.

Both PLS models were able to discriminate the cultivation system, in calibration and validation, with R^2^_v_ values of 0.74 and 0.60 for PLS-1 and PLS-2, respectively (Table 5). The PLS-1 outperformed the second model since the non-destructive method used allowed to make more measurements for each replication, which were more than the replications used in the analytical method. This is due to the rapidity of the analysis, which allowed us to analyze more samples, catching all the variability present among the samples. The possibility of giving information about the cultivation system to the final users could be useful for the growing awareness of modern consumers toward the economic, social, and environmental sustainability of production processes. Moreover, it represents another piece of information that might influence the purchase, since consumers are increasingly interested in knowing the history of the product. On the other hand, the cultivation system can affect some nutritional aspects and the quality of the product at hand.

## 4. Conclusions

The soilless system (ScS) allowed for better optimization of water use, with only slight differences noted with reference to the fertilization program adopted. Fertilizers PFP was by far higher in the case of low-input fertilization (LF) treatments, both in SbS and in ScS, allowing satisfying production levels and the more sustainable management of nutrients.

Postharvest qualitative analysis confirmed the efficiency of the LF treatment applied in ScS, resulting in a more environmentally sustainable approach, with positive effects also recorded for the postharvest quality of fresh-cut rocket leaves. In particular, lower accumulation in ammonia and reduced electrolyte leakage was measured in LF-ScS rocket leaves, meaning that they were of higher quality than HF treatment.

Finally, data obtained by the present study allowed us to build a PLS model able to predict the product history (the cultivation system), starting from the non-destructive analysis of total chlorophyll. This last result might be a valid tool for giving additional information to the users (consumers, logistics operators) regarding the cultivation technique used for wild rocket.

## Figures and Tables

**Table 1:** Chemical and physical soil properties in soil-bound sector.

Soil Properties	Unit	Value
Sand	%	24
Silt	%	32
Clay	%	44
Total-CaCO_3_	%	1.32
Organic matter	%	1.08
Organic carbon	%	0.99
Total-N (Kjeldhal method)	‰	1.06
pH	-	7.8
EC	dS/m	2.4
Exchangeable K (BaCl_2_^−^TEA)	mg/kg	264
Available-P_2_O_5_ (Olsen method)	mg/kg	98.7
Cation exchange capacity	meq/100 g	34.5

**Table 2:** Yield, dry matter; water use efficiency (WUE); N, K, P, Ca, and Mg partial factor productivity (PFP); and leaf chlorophyll content of rocket leaves cultivated via soilless cultivation system (ScS) with high input (HF) or low input (LF) of nutrients supplied as fertilizer solution.

Treatment	Yield	Dry Matter	WUE	PFP					Leaf Chlorophyll Content
				N	K	P	Ca	Mg	
	(g/Pot)	%	(g/L)	(g Yield/g of Nutrient Supplied)					µmol Chlorophyll/m^2^ (Leaf Surface)
LF	412	7.9	34.7	226	203	1158	273	869	370
HF	458	8.7	33.1	158	141	1066	165	689	378
Significance ^(1)^	***	***	**	***	***	***	***	***	ns

^(1)^ Significance: ns: not significant; ** and ***, respectively, for *p* ≤ 0.01 and *p* ≤ 0.001.

**Table 3:** Yield, dry matter, water use efficiency (WUE), N partial factor productivity (PFP), and leaf chlorophyll content of rocket leaves cultivated via soil-bound system (SbS) with high input (HF) or low input (LF) of N fertilizer.

Treatment	Yield	Dry Matter	WUE	N PFP	Leaf Chlorophyll Content
	(g/m^2^)	%	(g/L)	(g Yield/g of Nutrient Supplied)	µmol Chlorophyll/m^2^ (Leaf Surface)
LF	3909	9.3	11.9	217	435
HF	4265	9.7	13	118	454
Significance ^(1)^	*	*	*	***	ns

^(1)^ Significance: ns: not significant; * and ***, respectively, for *p* ≤ 0.05 and *p* ≤ 0.001.

**Table 4:** Effects of fertilization programs (high input, HF, or low input, LF), storage times (0, 6, 9, 12, or 16 days), and their interactions on sensory, physical, and chemical parameters of fresh-cut rocket leaves cultivated via soilless (ScS) or soil-bound (SbS) systems sampled at two harvest cuts (HC1 and HC2) and stored at 10 °C.

Parameters	ScS						SbS					
	HC 1			HC 2			HC 1			HC 2		
	Fertilization Program (A)	Storage Time (B)	A × B	Fertilization Program (A)	Storage Time (B)	A × B	Fertilization Program (A)	Storage Time (B)	A × B	Fertilization Program (A)	Storage Time (B)	A × B
Respiration rate (µmol CO_2_/kg s)	**	****	****	****	****	**	ns	****	ns	ns	****	ns
Visual quality (5–1)	ns	****	***	*	****	*	ns	****	ns	ns	****	ns
ΔE*	****	****	****	ns	****	ns	ns	****	ns	ns	****	ns
Hue angle (°)	ns	****	ns	ns	****	ns	ns	****	ns	ns	****	ns
Yellowness Index (YI)	ns	****	*	*	****	ns	ns	****	ns	ns	****	ns
Ammonia content (µg NH_4_^+^/g)	**	****	**	**	****	****	ns	****	ns	ns	****	ns
Electrolyte leakage (%)	****	****	ns	ns	****	****	ns	****	ns	ns	****	ns
Dry matter (%)	****	ns	ns	ns	ns	ns	ns	ns	ns	ns	ns	ns
Total chlorophyll content (mg/100 g fw)	***	****	**	ns	****	****	ns	****	ns	ns	****	ns
Antioxidant activity (mg Trolox/100 g fw)	**	****	****	ns	***	*	*	****	ns	ns	****	ns
Total phenols (mg gallic acid/100 g fw)	***	****	**	ns	***	ns	ns	****	ns	ns	ns	ns

Results are given as mean values of 50 samples (5 replicates × 2 fertilization levels × 5 storage times). ns: not significant; **** significant for *p* ≤ 0.0001; *** significant for *p* ≤ 0.001; ** significant for *p* ≤ 0.01; * significant for *p* ≤ 0.05.

**Table 5:** Root Mean Square Error (RMSE) and the coefficient of determination (R^2^) in calibration (c) or validation (v) of the partial least square regression (PLS) models predicting the cultivation system of rocket leaves.

PLS Models	Tools for Predictors	RMSE_C_	R^2^_c_	RMSEv	R^2^_v_
PLS-1	Apogee MC-100 (non-destructive tool)	0.25	0.76	0.26	0.74
PLS-2	Analytic methods (destructive tools)	0.30	0.63	0.32	0.60

## Data Availability

Data are contained within the article.
